# Significant others, situations and infant feeding behaviour change processes: a serial qualitative interview study

**DOI:** 10.1186/1471-2393-13-114

**Published:** 2013-05-16

**Authors:** Rhona J McInnes, Pat Hoddinott, Jane Britten, Kirsty Darwent, Leone CA Craig

**Affiliations:** 1 School of Nursing, Midwifery & Health, University of Stirling, Stirling FK9 4LA, UK; 2 Work carried out at the Health Services Research Unit; University of Aberdeen, 3rd Floor, Health Sciences Building; Foresterhill, Aberdeen AB25 2ZD, UK; 3 Division of Applied Health Sciences, Public Health Nutrition Research Group, Polwarth Building, Foresterhill, University of Aberdeen, Aberdeen AB25 2ZD, UK

**Keywords:** Infant feeding, Behaviour change, Qualitative, Person-centred care

## Abstract

**Background:**

Exclusive breastfeeding until six months followed by the introduction of solids and continued breastfeeding is recommended by the World Health Organisation. The dominant approach to achieving this has been to educate and support women to start and continue breastfeeding rather than understanding behaviour change processes from a broader perspective.

**Method:**

Serial qualitative interviews examined the influences of significant others on women’s feeding behaviour. Thirty-six women and 37 nominated significant others participated in 220 interviews, conducted approximately four weekly from late pregnancy to six months after birth. Responses to summative structured questions at the end of each interview asking about significant influences on feeding decisions were compared and contrasted with formative semi-structured data within and between cases. Analysis focused on pivotal points where behaviour changed from exclusive breastfeeding to introducing formula, stopping breastfeeding or introducing solids. This enabled us to identify processes that decelerate or accelerate behaviour change and understand resolution processes afterwards.

**Results:**

The dominant goal motivating behaviour change was family wellbeing, rather than exclusive breastfeeding. Rather than one type of significant other emerging as the key influence, there was a complex interplay between the self-baby dyad, significant others, situations and personal or vicarious feeding history. Following behaviour change women turned to those most likely to confirm or resolve their decisions and maintain their confidence as mothers.

**Conclusions:**

Applying ecological models of behaviour would enable health service organisation, practice, policy and research to focus on enhancing family efficacy and wellbeing, improving family-centred communication and increasing opportunities for health professionals to be a constructive influence around pivotal points when feeding behaviour changes. A paradigm shift is recommended away from the dominant approach of support and education of individual women towards a more holistic, family-centred narrative approach, whilst acknowledging that breastfeeding is a practical skill that women and babies have to learn.

## Background

There is considerable evidence to support the recommendation by the World Health Organisation (WHO) of exclusive breastfeeding (no other fluids or solids) for six months, followed by the appropriate introduction of solids and continued breastfeeding for two years and beyond [1,2]. Recommendations are founded on the short and long term health benefits for both infant and mother and consequently breastfeeding is a global health priority. Health benefits include reduced infant gastro-intestinal and respiratory infections, with consequent reduced numbers of hospital admissions; improved developmental and educational outcomes; and a reduced risk of maternal breast and ovarian cancers [2]. However, breastfeeding rates often fall short of this recommendation [3,4] for example in the UK around 1% of babies were exclusively breastfeed to six months in 2010 [3]. This rate has remained static for several years despite increases in the numbers of women initiating breastfeeding. The steepest drop off in breastfeeding is in the first few weeks of life and 86% of those who stop in the first two weeks would like to have continued for longer [3]. Evidence from qualitative research highlights that many women’s needs are not being met by health services [5] and a recent UK Infant Feeding Survey [3] indicates that care for effective breastfeeding is often insufficient, for example less than half of new mothers were informed about how to recognise that their baby was getting enough milk. Many women turn to their social network for help but in a society where formula feeding is the norm, experience, knowledge and confidence in breastfeeding is often low [5]. Systematic reviews indicate inconsistent effectiveness of interventions to maintain breastfeeding, with outcomes varying according to socio-economic status, baseline breastfeeding prevalence and context [6,7]. Prior to our study, we considered how behaviour change theory can help us to understand how this situation might be improved.

### How infant feeding fits with behaviour change theory

A review of lifestyle behaviour change interventions identifies inconsistent effectiveness of the more commonly applied psychological theories of behaviour and emphasises the importance of context, social factors and the social environment [8]. The evidence in this review is predominantly derived from behaviours very different from breastfeeding, which is a highly skilled behaviour that has complex physiological, hormonal, psycho-social and cultural mediators, therefore generalisability cannot be assumed. However, although interventions to improve breastfeeding outcomes have seldom made explicit use of behaviour change theory the following two themes which underpin research are important to consider. Firstly, interventions have tended to follow trends in health promotion for other lifestyle behaviours and secondly they have had a strong focus on providing support to women to prevent problems arising, and thus increase breastfeeding prevalence.

In the past thirty years, behaviour change interventions initially targeted knowledge of benefits (for example the Health Belief Model) with the assumption that women would rationally choose breastfeeding once they knew it was healthier. Later research focussed on attitudes as the precursor to intention in order to predict feeding behaviour, for example testing or applying the Theory of Reasoned Action [9]. These theories assume a linear and rational decision-making process usually in response to a perception of health threat or risk. However, our earlier data analysis identified infant feeding behaviour changes made at times of significant emotional distress, often as a crisis response rather than a coherent rational decision [10]. Many theories make limited reference to any discrepancy between intention and actual behaviour [11,12], for example, the Theory of Reasoned Action does not fully explain the correlation between intention and infant feeding behaviour without adding social support and subjective norms [13]. To some extent this discrepancy has been addressed, for example by including motivational and action stages such as in the Stages of Change Model [14], or by considering the individual’s perception of ability to control behaviour such as in the Theory of Planned Behaviour (TPB) [15,16]. Psychological models like TPB demonstrate that decision-making processes around breastfeeding are not always rational, but relate to other factors such as moral norms. Acknowledging breastfeeding as a skilled behaviour rather than a simple choice led to the application of Self-Efficacy Theory [17], which endures as an important construct for successful breastfeeding [18]. Although some behaviour change models reflect the complexity of infant feeding decision-making, women’s choices are not simply between health and risk but are inextricably linked with the concept of the ‘good mother’ [19,20]. Breastfeeding can be crucial to women’s identity as a mother and may also compensate for other senses of failure, for example arising from postnatal depression or a ‘failed’ birth [21]. In short behaviour theories often propose over simplistic assumptions around behaviour planning, particularly regarding stability and coherence of plans and conscious rational decision-making [22].

The concept of breastfeeding support has dominated and underpins most trials [6] with influence mediated by age, ethnicity, proximity and socio-cultural background [23,24]. Support was conceptualised by Sarafino [25] as instrumental; emotional; information giving; esteem and network and applied to breastfeeding [26,27]. But ‘support’ is a word used more often by professionals than women, who tend to ask for ‘help’, and can imply a breastfeeding-centred, promotional approach, which can be met with resistance and conflicts with woman-centred care [28]. The provision of support for breastfeeding recognises the importance of the context in which infant feeding is situated. Social network and behavioural norms have a strong influence on women’s feeding choices, outcomes and feeding efficacy [5]. Women from families or social networks where breastfeeding is the norm are significantly more likely to intend to breastfeed and to breastfeed successfully especially if they themselves have been breastfed [3].

Our aim was to investigate how parents and their significant others influence feeding behaviour change. This paper builds on earlier findings from a longitudinal qualitative interview study. The overall research aim was to explore the early infant feeding experiences of parents and their significant others during the first six months of life, and to answer the research question: what would make a difference? [29]. Initial findings reported that families, their social networks and the health service hold different philosophical positions of idealism or realism about infant feeding [10]. Pivotal points, where feeding behaviour changes away from the ideal of exclusive breastfeeding for six months, were often accompanied by a conflict between idealism and realism. The outcome that mattered to participants was maternal, baby and family wellbeing [10]. We set out to explore patterns of significant other influence before and after these pivotal points. Given the inconsistent evidence for individually tailored behaviour change interventions [7] our approach is informed by environmental and ecological theory, which understands health related behaviour as constantly adapting to changes in the micro, meso and macro context [30]. The micro-system is the face to face level of interactions in specific settings with friends, family, health professionals; the meso-system refers to the interrelations between the various settings that the individual is involved in, for example breastfeeding in hospital, at home, in public, at work; and the macro-system refers to the larger social, political and cultural environment in which the woman and her family are embedded [30].

## Methods

Serial qualitative interviews were chosen as trust can develop between the researcher and participant, which facilitates in depth exploration of how perspectives, experiences, relationships and behaviour change over time [31].

### Definitions

Breastfeeding initiation refers to the baby receiving any breast milk, even if only once. Exclusive breastfeeding is defined as the infant receiving only breast milk since birth with no other liquids or solids with the exception of drops or syrups consisting of vitamins, mineral supplements, or medicines [32]. Introduction of solids is defined as the first ever solid food offered to and taken by the baby, even if it is only a small amount and this includes solids that are liquidized as soups or purees. This is congruent with the World Health Organisation definition for complementary feeding, however in reality introduction of solids has a variety of meanings for families [29]. Pregnant women in the study are the index cases and relationships are described in relation to them. Significant other(s) is/are the person(s) identified by the woman who has the strongest influence on feeding decisions, regardless of the direction of influence (either for or against the decision). We use the term ‘woman-centred’ to indicate an approach which facilitates a woman’s own infant feeding decision-making and supports her in the choices she makes, focusing on her needs rather than solely on breastfeeding. ‘Breastfeeding- centred’ refers to an approach which prioritises the goal of continued breastfeeding above all else. A breastfeeding-centred approach can be perceived by women as ’pressure’ or inducing ‘guilt’ [29,33].

### Data collection and interviewing

We aimed to recruit disadvantaged women, who are least likely to breastfeed [3], from two geographically separate areas where the maternity units were working towards UNICEF Baby Friendly Accreditation [34]. Information packs were sent to 541 women due to give birth in September and October 2009. A sampling frame for the characteristics listed in Table 1 was used to select 18 women at each site from the 72 volunteers (13% of the invited sample) completing an opt-in questionnaire. Although more than 70% of our sample lived in the three more disadvantaged Scottish Index of Multiple Deprivation (SIMD) quintiles [35], women from the most disadvantaged quintile and younger mothers were under represented in our sample indicating that this was not a reliable method of identifying disadvantage. It is likely that the women who volunteered were among the more advantaged living in disadvantaged areas [29].

**Table 1:** Characteristics and feeding behaviour of women interviewed (n = 36)

	**Site 1 participants (n = 18)**	**Site 2 participants (n = 18)**
Age (years)		
≤20	0	3
21-30	4	4
31-40	11	11
≤40	3	0
Age at leaving full time education (years)		
16 or less	1	3
17	1	5
18	3	1
19 or more	13	9
Occupational classification^a^		
1-3	10	6
4-6	5	8
7-9	2	3
Not employed	1	1
Parity		
0	9	10
≥1	9	8
Scottish Index of Multiple Deprivation (SIMD)^b^		
1-3	13	13
4-5	5	5
Feeding:		
Exclusive breastfeeding to six months	1	0
Any breastfeeding to six months	10	7
Formula introduced in first week	7	9
Formula introduced by six weeks	11	13
Stopped breastfeeding by six weeks (including 1 who didn’t start)	3	7
Solids introduced by 20 weeks: n = 34 (no solids data for 2 Site 2 women)	7	13

^a^Standard Occupational Classification (SOC 2000) taken from the 2000 Census (Office of National Statistics, 2001):

1. Managers and senior officials.

2. Professional occupations.

3. Associate professional and technical occupations.

4. Administrative and secretarial occupation.

5. Skilled trade occupations.

6. Personal service occupations.

7. Sales and customer service occupations.

8. Process and plant and machine operatives.

9. Elementary occupations.

^b^Scottish Government, 2009. SIMD 1 is the most deprived quintile. SIMD 5 is the least deprived quintile.

Two researchers, (one at each site) interviewed women and asked them to identify significant others (partners, family, friends and health professionals) who might be interviewed. Researchers then obtained informed consent to interview a diverse sample of information rich significant others at different points. Twenty-six partners, eight maternal mothers, one sister and two health professionals nominated as significant others were interviewed. To minimize bias and researcher assumptions, a multi-disciplinary research team was configured to bring together considerable infant feeding research experience from different backgrounds: nutrition; the voluntary sector; social policy; midwifery and general practice.

Face to face semi-structured interviews took place at home during pregnancy, within six weeks of birth and at six months, with shorter, mostly telephone, interviews (0–5) in between. Interview frequency and method of contact was negotiated individually, with two participants preferring face to face interviews throughout as English was not their first language. In total, 220 recorded and fully transcribed interviews were conducted. Topic guides, modified through research team discussion throughout the study, were used to probe emerging themes and search for disconfirming data. Towards the end of each interview, participants were asked, ‘Who has had the strongest influence on your feeding decisions since we last spoke?’ Any qualitative and survey data enquiring about experiences may be influenced by post-hoc rationalisations and priming by earlier discussions. The four weekly serial interviews enabled both prospective data collection about future plans and expectations combined with retrospective reflections on the feeding journey which could then be compared during the data analysis and in research team meetings. For example, a woman who changed from breast to formula feeding commented that she had breastfed to prove that she could, not because she really wanted to, which was contrary to what she had stated antenatally.

Two structured information forms were completed: on significant other characteristics (age, relationship, distance from the family and feeding experience) and breastfeeding (duration, exclusivity and introduction of non-milk liquids and solids). Ethics approval was obtained from the North of Scotland Research Ethics Committee and further detail on sampling and data collection is available [29].

### Data analysis

Interview transcripts were entered into FrameWork software [36] and data collection and analysis progressed iteratively, with the four authors listening to interview recordings, reading verbatim transcripts, identifying and interpreting themes and agreeing modifications to topic guides according to the emerging analysis [29]. Four researchers independently constructed a thematic index by reading a sample of six information rich transcripts of antenatal and first postnatal interviews, then reached consensus through discussion, with the index modified later in a similar manner to cover the introduction of solids. A final thematic index was agreed approximately half way through data collection and was used to organise, label and thematically summarise data. Analysis proceeded by researchers keeping reflective diaries, identifying and discussing interpretive themes, generating research questions, creating different FrameWork charts to explore patterns and search for disconfirming data. Early charts compared primiparous with multiparous couples; early versus late breastfeeding cessation or introduction of solids [10]. Excel charts with structured data on the type, number, distance and infant feeding experience of significant others (self-baby dyad, health professional, partner, female friends and family) initially explored the relationships between women and significant others, however no clear patterns were identified.

We continued to explore significant other and wider social influences on feeding behaviour, using thematic analysis to understand the meanings of actions and interactions around behaviour change. We identified interviews conducted immediately before and after the three pivotal points where behaviour changed: a) the introduction formula feeding; b) stopping breastfeeding and c) the introduction of solid food. We used the constant comparative method to contrast themes within and between cases, comparing them with interviews where exclusive breastfeeding was maintained. Inconsistencies between the semi-structured interview data (covert influences on behaviour) and the structured data (overt influences on feeding behaviour), or changes in the person(s) nominated as significant around the three pivotal points were explored. Our findings present a synthesis of the structured and semi-structured data analysis focusing on before and after behaviour change. Similar syntheses for feeding decisions in pregnancy and for maintaining breastfeeding are available [29].

## Results

**Figure 1:**
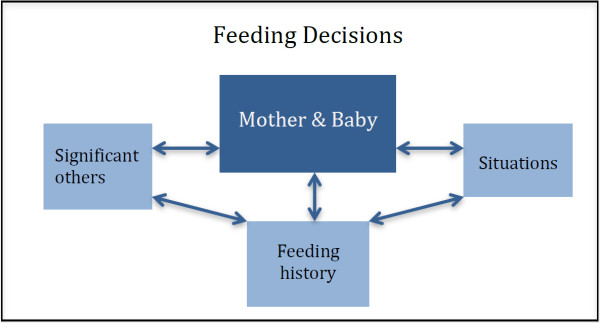
Influences on infant feeding.

**Figure 2:**
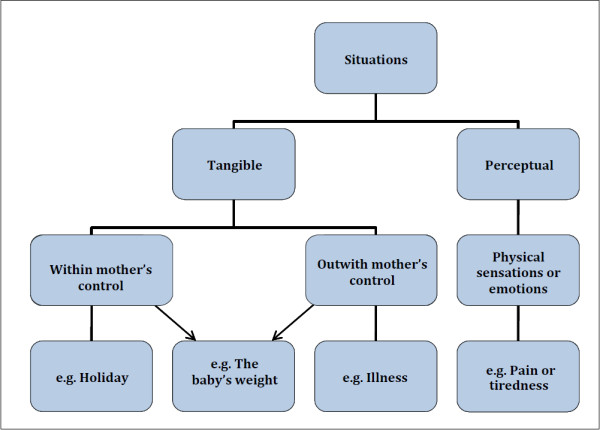
Situations.

**Figure 3:**
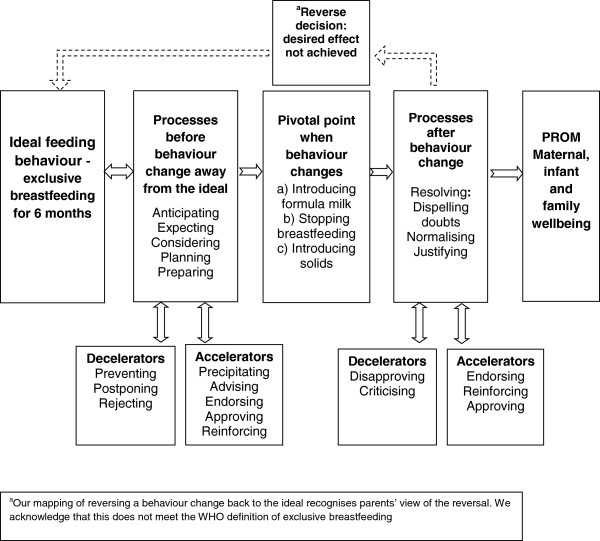
Feeding behaviour, influencing processes and the ultimate participant reported outcome (PROM) that matters.

Significant others, the self-baby dyad, situations and feeding history, were influential on decision-making and behaviour change (Figures 1 and 2) through a variety of influencing processes (Figure 3).

### Significant others and feeding behaviour

Significant others named by women at the end of interviews are summarised in Table 2. Overall, primiparous women named more significant others than multiparous women and the number of significant others named at each interview was often consistent within cases, with a decline in the number as the baby aged. There was considerable variation across cases ranging from a woman naming only herself throughout the study to a maximum of nine significant others. Significant others were not always those living closest; a trusting relationship often seemed to override distance, helped by the comparative ease of telephone and internet communication. Women whose first language was not English named significant others living abroad.

**Table 2:** **Significant others**^**a **^**named by women in structured data collection at the end of interviews (n = 36)**

	**Self/baby**	**Health profess-ional**	**Partner**	**Female network**			**Media/ culture**	**Male relative**
				**Mother**	**Friend /colleague/ group**^**b **^**member**	**Other female relative**^**c**^		
Primips (n = 19)	14	18	13	13	12	10	6	2
Multips (n = 17)	16	12	14	9	6	7	1	0
**Total (n = 36)**	**30**	**30**	**27**	**22**	**18**	**17**	**7**	**2**

^a^ All relationships relative to the woman.

^b^ Parent/baby groups or online baby/parenting forums.

^c^ Excludes the woman’s own mother.

Thirty women named one or more health professional as a significant other at some point, with primiparous women more likely to do so (18/30). One primiparous woman who named few significant others named health professionals at every interview whereas five multiparous women never mentioned health professionals. Midwives were named by 13 women, most frequently by primiparous women (9/13) and health visitors were named by 26 women, most often around introducing solids or with GPs (named by four women) in connection with situations such as lack of weight gain and mastitis. Some parents named themselves as a significant influence: ‘you have to be the one to initiate [formula feeding]’, as health professionals were perceived as not recommending this. Health professionals were more likely to be overtly named as influential when they endorsed women’s decisions.

Partners were overtly named by 27 women. There were differing expectations and experiences of couple roles with at one extreme a ‘share everything’ parenting style with fairly equal involvement in infant feeding and at the other women describing themselves as a ‘one man band’, making their own decisions. Partners who were involved in feeding might be named as a significant influence but were less likely to be named if their attitude to feeding differed from that of women or if any change they suggested was not successful.

Female network members were named by 29 women, particularly primiparous women, with mothers particularly mentioned around introducing solids. When women sought support from people ‘going through the same as you’, new friends, for example from the breastfeeding group, were named for the first time instead of older, formula feeding friends who said ‘just bottle feed her’.

### Self – self-baby dyad

A few women struggled with answering the structured summative questions about significant others, often concluding that they themselves were their main influence. Sometimes the response ‘self’ was given when a woman had listened to a range of people and had ‘picked the bits out of everybody’s that I’ve thought “well that fits in with what I’m thinking”’. Multiparous women who had previously breastfed and older women more often named themselves. Our interpretation is that women who cited themselves are indicating greater certainty and efficacy in their feeding decisions: ‘I haven’t needed help’. Their motivation to act may be intrinsic rather than located within their social network. Babies were named by four women as the significant influence at the first postnatal interview and later by eight when solids were being considered. The baby was named as a significant influence when problems with breastfeeding occurred, such as the baby not latching, wanting to feed ‘non-stop’, or failing to gain weight, when changing feeding seemed to be the only response in the circumstances. There was a continuum for baby influence, from overt baby-led feeding to the baby not being overtly named with women saying ‘it’s just me who makes the decisions’, but often with covert references to the baby’s influence during interviews.

Woman: ‘…I was going to give up the breastfeeding at six months anyway because I felt that would be enough. But I put her on [formula] two weeks ago now maybe, the reason being because she wasn’t taking the formula, so I was continuously trying her with the bottle and when she did take it I just put her on it just in case she wouldn’t take it again.’ (ID 2037. Interview 24 weeks after birth: formula introduced at 9–12 weeks, breastfeeding stopped and solids introduced at 21–24 weeks. Significant others: partner and self)

### The importance of the situation

Situations were important influences on feeding and were divided into tangible or perceptual (Figure 2). Tangible situations fell onto a continuum from those completely within maternal or parental control (changing feeding for a holiday or social activities) to those that were not (illness, parental leave, needs of siblings). Some situations such as baby’s slow weight gain may or may not be within parental control. Perceptual situations often related to physical sensations or emotions arising from feeding, such as pain, anxiety and lack of sleep, but also from growing confidence with feeding, enjoyment and the ability to relax. Perception of the baby’s needs also influenced behaviour, for example when the baby appeared ‘hungry’, woke more at night or constantly watched others eating. There are complex dynamic relationships between the mother, the baby, named significant others (overt influences), tangible and perceptual situations, for instance a woman may mention her baby as a significant other but the tangible situation of having a baby weighed might trigger feeding behaviour change.

### Feeding history

Feeding history refers to women’s own experiences of feeding babies and how couples were themselves fed. Primiparous women in particular described a vicarious feeding history based on stories from family and friends, antenatal education and the media, especially the internet.

Previous experience of feeding was a very strong influence for multiparous families and two patterns were observed. Firstly, doing ‘the same’ as with an older child, because of familiar routines, previous successful feeding or an obligation to feed this baby the same way: ‘I’m wanting to try and keep it up, because [older child] was breastfed for six weeks.’ Secondly, trying a different method of feeding either to meet the needs of older children or because previous experience did not meet the family ideal for feeding and/or wellbeing. Twenty-six women had themselves been breastfed as babies and as reported elsewhere they were more likely to continue breastfeeding [3].

### Influencing processes for feeding behaviour change

The focus of our thematic analysis changed from ‘who’ or ‘what’ to ‘how’ behaviour change was initiated. Emerging from our data were influencing processes that unfolded before, during and after a change (Figure 3). Influencing processes could emerge gradually, with anticipation of future feeding change, or could be more actively considered, for example weighing up the pros and cons, planning or preparing for change. Women described situations that accelerated or decelerated change. Breastfeeding difficulties such as pain in the early weeks could rapidly precipitate very intense pivotal points with quick decisions and actions. By contrast, a more gradual build up to a less intense pivotal point was common around the introduction of solids. Families own emergent thinking about a decision could be reinforced, endorsed or approved by others, by feeding history or by situations, for example thinking about introducing formula could be reinforced by the baby’s poor weight gain and family comments based on their experience.

The process themes described in Figure 3 differ subtly in how they reflect women and families’ underlying certainty, confidence, commitment and values. These processes formed a dynamic continuum of influence, rather than static categories, with considerable change forwards and/or backwards over varying timescales. Similarly processes that helped to resolve a behaviour change without loss of confidence or self-esteem, for example endorsing and approving were often continuous over a pivotal point.

The following sections describe how the processes by which significant others, situations and feeding history influence behaviour change were derived from the data.

### Decelerators of feeding behaviour change

Significant others and situations could postpone behaviour change overtly or covertly particularly around the introduction of solids and occasionally formula. Partners who prevented behaviour change were those who did not want to personally feed the baby but provided ‘great support by looking after the kids and running after the house’ or kept ‘reminding me of the benefits’ allowing women to focus on breastfeeding. Similarly mothers and mothers-in-law were overtly named or covertly described as preventing or postponing change by providing practical help, ‘filling the freezer’, hoovering and washing. Health professionals prevented change through care that was accessible and woman-centred, understanding and respecting women’s views. Pro-active practical help with breastfeeding was particularly valued. Communication styles which encouraged and helped women to feel confident were appreciated, when health professionals ‘show interest in the baby and make me feel important’, ‘always listen’, and take time.

Woman: ‘I can't sing her [health visitor’s] praises enough, … she's always open to listen to anything really, and she will not be negative if, for instance, I’ve said, “I don’t really know how much longer I can feed him” or whatever, and she’ll just chat with you, you know, she won’t sort of preach to you kind of thing. She’s really good.’ (ID 2192. Interview 10 weeks after birth: breastfeeding with formula introduced at 5–6 weeks. Significant others: health visitor)

Women expected health visitor’ influence around the introduction of solids, however contact was variable and interactions could either sustain breastfeeding through preventing or postponing change or could precipitate change ‘depending on who you see’. Discourses around these interactions were often linked to specific situations, particularly weighing the baby, poor sleep or an unsettled baby.

Older more educated women, were often keen to ‘follow the guidelines’ and situations such as the baby ‘putting on loads of weight just being breastfed’ supported ‘holding off’. Some women felt ‘it’s kind of selfish to put him on to food quite early just so I can get a night’s sleep’ and adopted a strategy of postponement by introducing more breast or formula feeds, or hungrier baby milk. Going on holiday and not wanting to take food, bowls and spoons, also led to postponement. Women’s confidence and/or previous breastfeeding experience were important and could mitigate a potentially negative situation like a baby’s admission to hospital, or a woman’s mastitis, which could trigger the end to breastfeeding for an unconfident first time mother. Rejecting behaviour change was most apparent in the accounts of women who named themselves or the baby as the strongest influence on feeding, or those drawing on experience with other children. These women expressed confidence and commitment to breastfeeding, had sometimes breastfed successfully before, or were finding breastfeeding ‘easy’, with a baby who ‘took to the breast like a pro’. Confident women rejected suggestions for changing feeding behaviour from partners who said, ‘is breast milk enough?’ or ‘just give him a bottle’, or from health professionals who were concerned about the baby’s health.

Woman: ‘they just said to me, “put him on formula feed” because they didn't think it would be successful for me to feed him with his weight loss, and I didn’t want to do that. … when they came back at 23 days one of the first things they said was, “did you put him on formula?” and I said, “no”… But they were overjoyed that he’d put weight on, so I think they realised that my decision was good.’ (ID 2295. Interview 4 weeks after birth: exclusive breastfeeding. Significant others: self and health visitor)

### Accelerators of feeding behaviour change

Often women anticipated the possibility of stopping breastfeeding to solve problems or to achieve the widely desired perceptual situation of ‘feeling in control’ and ‘getting into a routine’. They might identify with those who had already made changes: ‘I can understand why some people wouldn’t have the patience for it’.

Partners’ views or women’s perceptions of their partner’s views and needs were an important precipitant of behaviour change. Some partners wanted to be involved in the ‘special relationship’ or to feed the baby ‘to make the experience complete’. ‘Share everything’ couples were keen for both be involved in feeding, not wanting partners to ‘be left out’, with partners wanting to take some of ‘the strain’ or ‘make meals for us and puree a bit for the baby’. Likewise some women wanted their partner to have ‘more responsibility’ and hence bond with the baby, and spoke of the benefit of gaining some ‘freedom’ through expressing milk or using formula.

Unpleasant perceptual situations like breast pain triggered feeding change and partners did not want to see the mother ‘upset’ or ‘running on empty’. Change could be accelerated if friends and family advised not ‘jumping over hurdles to give the baby breast milk’, or expectations of help needed to maintain breastfeeding were not met: ‘someone to look after me, a mummy’.

Early postnatal accounts of situations and interactions with health professionals frequently described emotional distress: ‘pressure’, ’upset’ and ‘stress’, which precipitated considerable behaviour change. Families commented on health service organisation and performance, especially staff being ‘too busy’, leading to missed opportunities for feeding help, which might have prevented behaviour change. Many would have liked more expert help to learn and sustain breastfeeding.

Woman: ‘the auxiliary nurse didn’t have experience, she didn’t have the patience and she really didn’t quite know what she was doing and she was just telling me what to do, she wasn’t, you know, showing me what to do… and that just basically ruined the whole thing….’ (ID 1044. Interview 3 weeks after birth: formula introduced and breastfeeding stopped in 1^st^ week. Significant others: partner)

Women described feeling isolated in hospital without partners, who ‘don’t get to share or support you in any part of it’, and the environment emerged as a key situation influencing feeding behaviour; for example a longer hospital stay, advised ‘to get breastfeeding established’ conflicted with a woman’s desire for ‘getting out’ to the ‘comfort of my own home’ and precipitated the introduction of formula. Health professional advice reduced emotional distress when it ‘confirmed’ women’s intentions to change feeding and came from a trusted health professional, often referred to by first name or as ‘my health visitor’.

Woman: ‘it’s been helpful when I’ve sort of said to them [health visitors] about him not settling and that I thought he was still hungry, it was quite good that they said, “yes, you feel he’s hungry then give him a bottle, that’s fine”.’ (ID 1075. Interview 8 weeks after birth: breastfeeding, with formula introduced in 2^nd^ week. Significant others: partner, health visitor and friend)

Health professionals named as a significant influence were often those who gave permission to ‘do what’s right for you’ and had a positive effect on confidence and wellbeing, consistent with woman-centred care.

Anticipating, expecting and preparing for change were common in relation to the inevitable introduction of solids when parents often looked forward to progress: ‘the next thing to think about is weaning’. Narratives described a wide range of tangible and perceptual situations precipitating the introduction of solids: family members and friends saying ‘give her food’; parents wanting more unbroken sleep; previous experience; and trying solids viewed as entertainment, seeing ‘how the baby reacts’ or copes with new tastes. Mothers and mothers-in-law were more likely to endorse introducing solids before six months in line with their practice and could accelerate change but could be ignored as being from ‘40 years ago’ when ‘things were a bit different’.

### After behaviour change

The processes of endorsement, approval and justification often spanned before and after the pivotal point when behaviour changed and helped families to resolve the change, enhancing self-esteem, confidence and wellbeing. Where significant others were disapproving or criticising, resolution was delayed or did not occur – engendering feelings of failure, guilt or remorse. Women turned to significant others to dispel doubts or justify feeding changes in the same way they sought endorsement and approval beforehand. Women wanted someone to say, ‘you’ve done the right thing, that’s fine’. They named significant others whose views matched their own, or who were ‘doing the same thing as me’, in preference to those whose advice differed. Professional endorsement of behaviour change was particularly valued when it helped to justify and resolve behaviour change, for example referring to a formula feed given at a particularly difficult time as a ‘crisis bottle’ and using a woman-centred approach ’it’s up to you’. Partners could justify a change: ‘I don’t want to give my baby formula milk in the first six months of his life, but actually it’s okay… we agreed’. The partner actively bottle feeding the baby sometimes resolved the change with a shift in values evident towards the importance of partners’ bonding and away from the theoretical longer-term benefits of exclusive breastfeeding. Our interpretation is that sharing feeding may provide a socially acceptable resolution to behaviour change which counteracts the stigmatisation and feelings of failure around early breastfeeding cessation.

Woman: ‘They [my partner and my mum] were good, they supported me and said, ‘Well at least you did it to begin with’, and also from my partner’s point of view, I think he was kind of like in some ways a little pleased [when breastfeeding stopped] just because he got the chance to feed her as well.’ (ID 1167. Interview 19 weeks after birth: formula, introduced in 1^st^ week, breastfeeding stopped at 3–4 weeks. Significant others: partner, health visitor, mother, mother-in-law, women at baby groups)

Hearing about feeding experiences and situations similar to their own normalised and reinforced the behaviour change enabling women to feel ‘less guilty’. Consistent with the rosy idealistic portrayal of breastfeeding [10], women noticed that stories of mixed feeding seemed more prevalent after the behaviour change and were a revelation to some who realised ‘the amount of people who offer a top-up bottle’. Women described how changing to formula feeding helped them to establish routines and ‘feel more confident’ and in control, which justified the change. Resolution occurred if the outcome was a happy, ‘thriving’ baby who had gained weight on formula or solids perceived as ‘what she’s [the baby] needed’.

Woman: ‘I’m still disappointed it didn’t work out. But he’s putting on weight well and he’s happy, he’s growing well, so … and he’s now at the stage that we’re getting smiles and we’re getting a bit more interaction, so as long as he’s healthy, I guess that’s the main thing.’ (ID 2061. Interview 9 weeks after birth: formula, introduced in 1st week, breastfeeding stopped 5–6 weeks. Significant others: baby, health visitor, GP)

Couples occasionally described people who were critical of a feeding change. Partners who said, ‘why do you want to give her formula milk?’ or ‘I’m not keen, I don’t even like the smell of the stuff’. Friends who followed what they perceived as the feeding rules [10] sometimes disapproved when women broke them and a breastfeeding-centred health visitor might say, ‘oh dear’ on hearing that breastfeeding had stopped or solids started. When the feedback was not what women wanted to hear, resolution could be achieved by avoiding health professionals, who ‘aren’t human about it’, ‘not listening’. Occasionally parents withheld information, with evidence of self-reliance: ‘doing it myself’ or keeping the introduction of solids ‘a guilty secret’.

Partner: ‘I was giving him [toddler] some dinner at three and a half months…and I never telled anybody, until it was months later and we realised everything was alright, because I … knew I’d get criticised, “Oh no, it’s four months”.’ (ID 2287. Interview 24 weeks after birth: formula, introduced at 3–4 weeks, breastfeeding stopped at 7–8 weeks, solids introduced at 16 weeks or less. Significant others: self and partner)

The decision to introduce formula or solids might be reversed, for instance a mother who was advised to ‘give him a bit of formula just to give him something to eat’ who then reverted back to breastfeeding: ‘Monday morning it was a completely different baby’. Or when the desired effect was not achieved following formula or solids introduced to deal with difficult situations, such as a lack of sleep or frequent feeding. Babies behaviour could suggest they ‘did not like it’, or they became constipated, or the effect of baby rice was to ‘actually wake her up’. The burden of feeding if a partner was away could make it difficult to continue preparing solids, and lead to the decision being reversed.

## Discussion

This serial interview study provides new insights into the dynamic combination of people, situations and feeding history that influence feeding behaviour change and its resolution. It identifies accelerating and decelerating processes before and after behaviour change, which affect both the speed and direction of change and how it is resolved. Our earlier analysis of this data found that feeding behaviour is driven by the goal of current maternal, baby and family wellbeing rather than the policy ideal of exclusive prolonged breastfeeding to maximise future health gain. The emotional distress associated with feeding difficulties, particularly in the early postnatal period, is a strong precipitant for behaviour change and our data indicated that women often did not get evidence based or effective support from informed health professionals. The importance to women and families of resolving their feeding decisions to improve their emotional wellbeing by turning to people who endorse, approve and normalise their decision has received little attention to date. Women turn to health professionals who are woman-centred rather than breastfeeding-centred to help resolve guilt and any perceptions of not being a ‘good mother’. Socially accepted narratives are recounted, like the importance of partners giving a bottle or solids to share the special feeding relationship and improve bonding. Such narratives help to resolve women’s feelings of breastfeeding failure and justify their need for time out of the situation to improve family wellbeing. Influences and perceptions following behaviour change are important because these affect the stories that are told within social networks, across generations and influence how women feed subsequent children.

Our findings build on research that reports that infant feeding decision-making and behaviour are not simply a choice between health and risk, or a planned behaviour [37,38]. Feeding is inextricably linked with the concept of the ‘good mother’ [19] and successful breastfeeding can build confidence and self-esteem that may compensate for other senses of failure like postnatal depression or a ‘failed’ birth [21].

Increasing a woman’s self-efficacy and confidence are dominant discourses in the breastfeeding literature [18,39], whereas our data suggest that couple, family, and parenting efficacy might more accurately conceptualise the complex interactions. In our study, self-efficacy was more evident amongst women who had previously breastfed or with a social network where breastfeeding was the norm.

The mother-infant ‘bond’ has been strongly promoted as a goal to encourage women to breastfeed. Such promotion may be counterproductive if it neglects to mention how the father and other family members can ‘bond’ with the baby other than through active involvement in feeding [40,41]. A partner’s lack of opportunity to bond may be perceived as a threat to family wellbeing. This is particularly so for women who adopt a relationship-centred approach to parenting health behaviours, such as interdependence and communal coping [42]. Lewis et al. [42] suggest that behaviour change occurs when couples think about their relationship and co-operate to achieve positive outcomes rather than undermining one another’s efforts, with a shift from being mainly self-centred to seeing health outcomes as meaningful for the couple. This resonates with our findings where the dominant goal driving behaviour was couple, other children and wider family wellbeing, particularly when feeding anxiety, pain and distress posed a threat to couple relationships and to each other’s wellbeing. Others illustrate how breastfeeding may cease if family welfare is being harmed or it conflicts with other family demands [39,43].

Health professionals have considerable potential to become significant influences when families are reconciling feeding decisions with overall family wellbeing, and improved training in communication skills and effective breastfeeding care is recommended. Our findings support the current evidence for woman-centred care [28] and suggest extending it to family-centred care. Barriers to this are heavy postnatal workloads, inflexible structures and routines, with fixed time points in the UK for infant assessments and transition of care from midwife to health visitor. In addition, a rules based approach that discourages mixed feeding [10] and does not facilitate reversal of decisions once formula has been introduced or breastfeeding stopped is counterproductive. For positive narratives of breastfeeding experience to cascade through social networks, current postnatal feeding care needs to be more flexible to provide skilled help at pivotal points for behaviour change. Reconfiguring postnatal care to maximise the potential for health care providers to influence family wellbeing and feeding outcomes is required.

Goals and goal setting are important motivators and mediators for all lifestyle behaviour change [8]. Control Theory [44] suggests that behaviour is adjusted to meet a goal but if the discrepancy between current position and goal is too great or there is a lack of skills, motivation or strategies then the person may give up on their goal. If the goal that matters to parents is current family wellbeing, this might explain why breastfeeding intervention trials and the considerable promotion of breastfeeding have been disappointing in terms of breastfeeding prevalence and duration [7]. Many interventions and practices are underpinned by over simplistic, linear models educating or supporting women to breastfeed. Over simplistic, low intensity interventions are likely to miss the pivotal points when help might make a difference and have marginal impact on families who are struggling with complex, distressing issues impacting on family wellbeing. There is an urgent need to take a more holistic and ecological approach [30] to improving infant feeding outcomes by ensuring postnatal care, research intervention design, and wider community and policy are congruent with family wellbeing goals. Interventions need to move beyond the micro-level, from self-efficacy to family efficacy; span the meso-level including the settings where infant feeding occurs and integrate the power that macro-level policy interventions such as The Breastfeeding etc. Scotland Act [45] can have in shaping social discourses, as exemplified by the effectiveness of banning smoking in public places on smoking related hospital admissions [46].

### Strengths and limitations

Serial interviews with in-depth narrative accounts close to the time of feeding behaviour change is a study strength, together with triangulation provided by more structured data collection. However, the trusting relationship between the interviewer and interviewee can make it more difficult for the interviewer to maintain distance and neutrality over time. To counteract this, two members of the research team had no contact with participants. Study rigour was increased by having researchers from different backgrounds collecting data in two different locations and this aided the search for disconfirming data [47]. As with all qualitative or survey research which collects data on experiences, post-hoc rationalisation can occur. A strength of our study was our ability to prospectively ask about plans and expectations, and being able to compare and contrast these with later interview accounts and behaviours. This increased awareness of post-hoc rationalisation, which we discussed as a research team. Every effort was made to ask open questions, but even so, priming from previous interviews inevitably occurred and will have influenced the data collected. This was noted particularly when asking about significant other influences.

The question asked about ‘who’ had the strongest influence on feeding decisions illustrates the researchers’ a priori assumption that the most important influences would be people. In retrospect, re-framing this as ‘who or what had the strongest influence?’ or ‘how were you influenced?’ would have produced data reflecting the complexity of influence that emerged, but might not have revealed the important changes in significant other relationships from before to after a feeding change. Some participants had difficulty articulating the thought processes around decision-making suggesting that automatic, non-cognitive decision-making was occurring [48]. The presence of significant others at face to face interviews, is likely to have affected how the ‘who’ question was answered but there were examples where the significant other present was both named and not named as influential. Women were recruited to the study via a letter on maternity unit headed paper, which may have influenced data collected on health professional influences.

## Conclusions

Situations and the environment in which they occur, significant others and personal or vicarious feeding history all contribute to family processes which accelerate or decelerate feeding behaviour change. Health service structure, organisation and practice can impede timely health professional access and influence around pivotal points when infant feeding changes. The resulting infant feeding narratives influence social networks which span family generations and hold the key to understanding how health services might influence feeding behaviour. A paradigm shift is recommended away from linear models of support for breastfeeding which target individual women, towards a more family-centred narrative approach which builds family efficacy and confidence, whilst acknowledging that breastfeeding is a practical and performing skill that women and babies have to learn.

## Competing interests

The authors declare that they have no competing interests.

## Authors’ contributions

RM: substantial contribution to the design of the study, data analysis and interpretation; drafted and revised the manuscript and approves final version. PH: substantial contribution to the conception and design of the study, data analysis and interpretation; involved in drafting and revising the manuscript and approves final version JB: contribution to the study design and substantial contribution to data collection, analysis and interpretation. Involved in drafting and revising the manuscript and approves final version. KD: Contributed to the interpretation of data, was involved in revising manuscript and approves final version. LC: contribution to the study design and substantial contribution to data collection, analysis and interpretation. Involved in revising the manuscript and approves final version. All authors read and approved the final manuscript.

## Pre-publication history

The pre-publication history for this paper can be accessed here:

http://www.biomedcentral.com/1471-2393/13/114/prepub
